# On the mobility, membrane location and functionality of mechanosensitive channels in *Escherichia coli*

**DOI:** 10.1038/srep32709

**Published:** 2016-09-06

**Authors:** Jonas van den Berg, Heloisa Galbiati, Akiko Rasmussen, Samantha Miller, Bert Poolman

**Affiliations:** 1 Department of Biochemistry, Groningen Biomolecular Sciences and Biotechnology & Zernike Institute for Advanced Materials, University of Groningen, 9747 AG Groningen, The Netherlands; 2 School of Medicine, Medical Sciences and Nutrition, Institute of Medical Sciences, University of Aberdeen, Foresterhill, Aberdeen AB25 2ZD United Kingdom

## Abstract

Bacterial mechanosensitive channels protect cells from structural damage during hypoosmotic shock. MscS, MscL and MscK are the most abundant channels in *E. coli* and arguably the most important ones in osmoprotection. By combining physiological assays with quantitative photo-activated localization microscopy (qPALM), we find an almost linear relationship between channel abundance and cell survival. A minimum of 100 MscL (or MscS) channels is needed for protection when a single type of channel is expressed. Under native-like conditions MscL, MscS as well as MscK distribute homogeneously over the cytoplasmic membrane and the lateral diffusion of the channels is in accordance with their relative protein mass. However, we observe cluster formation and a reduced mobility of MscL when the majority of the subunits of the pentameric channel contain the fluorescent mEos3.2 protein. These data provide new insights into the quantitative biology of mechanosensitive channels and emphasizes the need for care in analysing protein complexes even when the fluorescent tag has been optimized for monomeric behaviour.

Biological membranes are complex structures allowing for the co-existence of single proteins and multiprotein assemblies in a milieu made of many different classes of phospholipids. Controlled localization of membrane proteins can be an essential aspect of cell growth and cell division^1^. Some proteins are present in the concave regions of the cell membrane, whereas other distribute homogenously over the lipid bilayer^2^,^3^. Membrane proteins can be organized in specific lipid domains and associate to form supramolecular structures^4^. Protein mobility has biological importance in membrane organization, protein-protein interactions, energy conversion, signalling, cell division, chemotaxis, and osmotaxis^5^,^6^,^7^,^8^. Here, we focus on understanding the localization, mobility and role in osmoprotection of the multisubunit complexes that make up the families of mechanosensitive (MS) channels. *E. coli* has seven MS channels, MscL (~75 kDa) being the most extensively studied one and the main contributor to cell survival during a hypoosmotic shock^9^,^10^. The other six channels belong to the MscS family, of which MscS (~210 kDa) and MscK (~900 kDa) are considered the major ones among the homologues^11^,^12^. When cells face a hypoosmotic shock MS channels act as emergency valves and they release solutes in a non-specific manner enabling a rapid decrease in osmolyte concentration and, consequently, in the osmotic driving force for water entry^13^. Mechanosensitive channels are interpreters of membrane tension, caused by changes in water movement across the membrane, and they gate in response to this signal. The interaction of MS channels with phospholipids has been studied using electrophysiology, crystallographic, spectroscopic and mutational analysis^14^,^15^,^16^,^17^,^18^,^19^,^20^,^21^,^22^,^23^,^24^,^25^,^26^. Despite great advances at the structural and functional level there are contrasting reports about the location of the channels in the membrane^27^. It is unclear if channels are organized in clusters^28^ and how many channels are required for osmoprotection.

To understand the dynamics of MS channels we combined single-molecule techniques with physiological assays. Photo-activated localization microscopy (PALM) and single-particle tracking (SPT) were used to study the membrane localization and mobility of channels, which were fused to the photo-switchable fluorescent protein mEos3.2. We found that MscS, MscL and MscK are mobile along the cell but that excessive overexpression of MscL-mEos3.2 led to cluster formation and retarded mobility, yet the channels are fully functional as shown by electrophysiology studies. Co-expression of wild type subunits with those tagged with mEos3.2 reduced the clustering, and thus we attribute the clustering to self-association of the fluorescent moieties rather than MscL itself. By combining quantitative PALM (qPALM) with a downshock assay we correlate the average channel numbers per cell with survivability after a rapid 0.3 M NaCl hypoosmotic shock. We find that around 100 plasmid-derived MscL channels confer full protection in a strain deleted for all chromosomal genes of MS channels. For MscS we could not achieve a condition to provide full osmoprotection but cell survival correlated with an increasing number of channels.

## Results

### Functional expression of fluorescent protein-labelled MS channels

**Figure 1:**
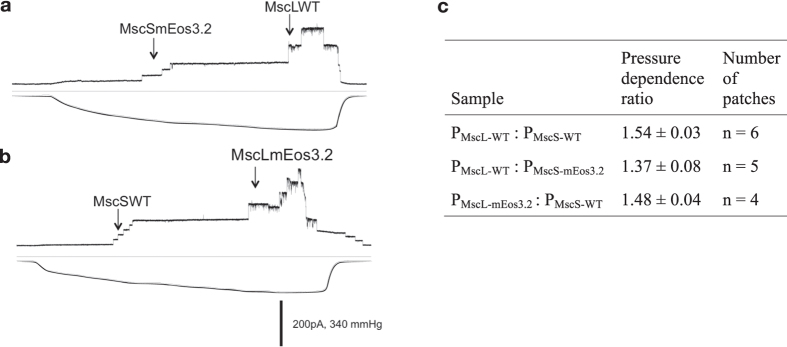
Patch clamp analysis of mEos3.2 tagged channels, benchmarked against the wild type proteins. Protoplasts prepared from (**a**) pBAD-MscS-mEos3.2 plasmid transformed into *E. coli* MJF429 (Δ*mscS* and Δ*mscK*) and (**b**) pBAD-MscL-mEos3.2 in *E. coli* MJF453 (Δ*mscL* and Δ*mscK*). We had an average number of MscS channels of 11 ± 9 which was estimated from various recordings; the numbers ranged from 2 to 24 “active” channels per patch (n = 7). The variations are so large that interpretation of the average number of channels per cell on the basis of patch clamp analysis seems questionable, and that is why we performed qPALM to quantify the number of MscL and MscS per cell. The arrows indicate the opening of the channels represented. (**c**) Channel activity determined by patch clamp electrophysiology. The pressure ratios were determined from patch clamp measurements in giant protoplasts made from *E. coli* MJF429 (Δ*MscS*, Δ*MscK*; *MscL* on chromosome) that had been induced with 1 mM IPTG for 15 min to express pTRC-MscS or 0.5% L-arabinose for 65 min to express pBAD-MscS-mEos3.2; this yielded the P_MscL-WT_ : P_MscS-WT_ (see also ref. 29) and P_MscL−WT_ : P_MscS-mEos3.2_ ratios. We used *E. coli* MJF453 (Δ*MscL*, Δ*MscK*; *MscS* on chromosome) that had been induced with 0.5% L-arabinose for 65 min to express pBAD-MscL-mEos3.2; this yielded the P_MscL-mEos3.2_ : P_MscS-WT_ ratio. The pressure ratios are given as mean ± standard deviation, and n represents the number of patches obtained to determine the pressure ratios.

Initially, pTRC-based vectors were created to express MscS, MscL and MscK with a C-terminal mEos3.2 and a His_6_-tag from the *trc* promoter (IPTG inducible). We determined the activity of wild type and labelled channels in *E. coli* cells by measuring viability after a 0.3 M NaCl downshock. MscS and MscL fusion proteins increased survival of *E. coli* MJF641, a strain that lacks all seven MS channels. The protection afforded by mEos3.2-tagged channels was slightly decreased relative to wild type channels (Supplementary Fig. S1), which likely reflects differences in expression levels as indicated by Western blots. We therefore performed patch clamp analysis to record the activity of individual channels. We found measurable activities for both MscL-mEos3.2 and MscS-mEos3.2, and the conductance and relative gating tensions of the tagged channels were similar to those of the parental proteins^29^ (Fig. 1).

### MscS, MscL and MscK are evenly distributed over the membrane

**Figure 2:**
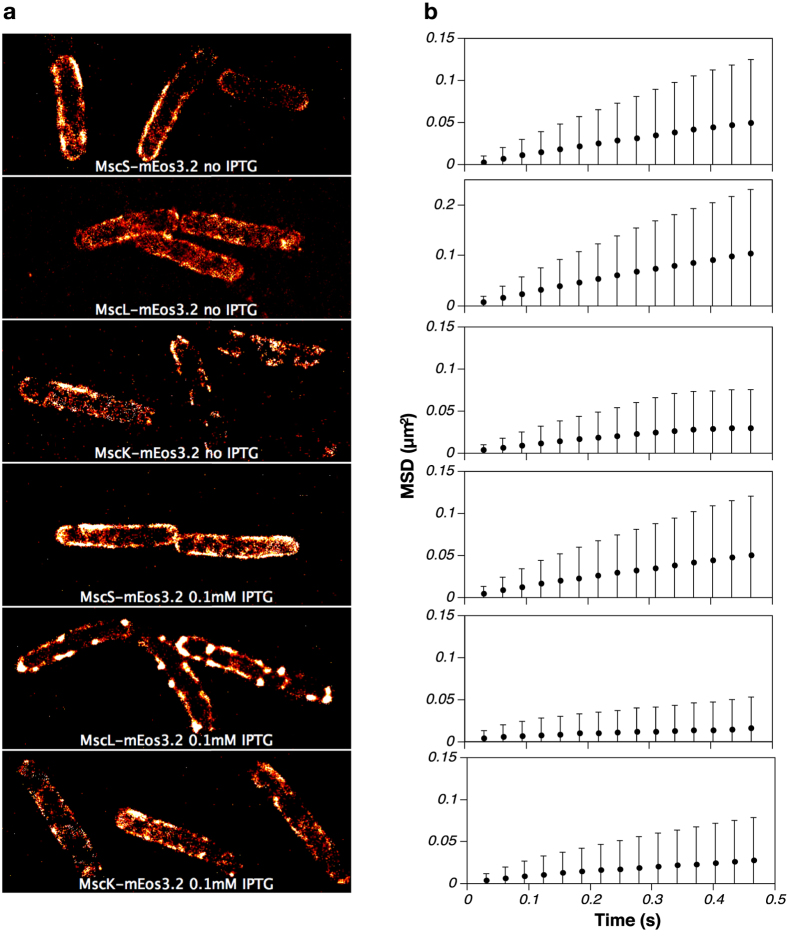
Distribution of fluorescently labelled mechanosensitive channels observed by PALM (**a**) Membrane localization of MscS-mEos3.2, MscL-mEos3.2 and MscK-mEos3.2 in *E. coli* MG1655 with and without 0.1 mM IPTG (30 min induction). (**b**) Mean square displacement plots of trajectories determined by single-particle tracking. Panels (b) correspond to the conditions shown in panel (a). On average about 10,000 trajectories were analysed. Error bars indicate the standard deviation.

We used PALM to reconstruct images of live *E. coli* MG1655 (non-fixed) in the exponential phase of growth and expressing MscS, MscL or MscK fusions with mEos3.2. In this strain chromosomally expressed MS subunits can mix and co-assemble with the respective plasmid-encoded mEos3.2 variants, creating channel complexes with different stoichiometries of non-tagged and mEos3.2-tagged proteins. With basal expression (no inducer) we observed random distribution of individual MS channels throughout the membrane (Fig. 2a; Supplementary Figs S2a and S3a). We did not observe a preference for pole localization for any of the MS channels tested. Similar data were obtained when cells were induced with 0.1 mM IPTG for 30 min (Fig. 2a; Supplementary Figs S2b and S3b), except that cells expressing high levels of MscL-mEos3.2 showed spots of higher intensity along the membrane, which we attribute to clustering of MscL channels.

### Cluster formation coincides with reduced mobility of MscL

To analyse the dynamic behaviour of the channels we performed single-particle tracking (SPT) of the mEos3.2-tagged proteins. This allowed us to track individual molecules for several time steps and to determine the lateral diffusion coefficients. The mean square displacement (MSD) of a membrane protein relates to the lateral diffusion coefficient *D* according to *MSD*(*t*) = 2*dDt*, with *d* being the dimensionality (*d* = 2). For the samples shown in Fig. 2a, the MSD was plotted (Fig. 2b) and the diffusion coefficients are shown in Table 1. MscL, MscS and MscK complexes have different masses and (predicted) radii of the membrane-embedded domains, which lead to different mobilities. The observed diffusion coefficients follow the radii of the complexes with MscL being fastest and MscK slowest. Increased expression did not significantly affect the measured mobility of MscS and MscK. In contrast, induction of MscL-mEos3.2 resulted in a roughly 10-fold reduced mobility (Table 1), which coincided with the cluster formation observed by PALM (Fig. 2a, Supplementary Fig. S3b). The fluorescence signal obtained from MscL-mEos3.2 upon induction originated both from the bright spots (large clusters of channels) and also from smaller clusters or single channels. Trajectories of individual MscL channels diffusing in the membrane (basal expression) and organized in clusters (overexpression, IPTG induction), obtained from single-particle tracking, are shown in Supplementary Video S1 and S2, respectively.

### A cumulative distribution function shows heterogeneous diffusion of MscL

**Figure 3:**
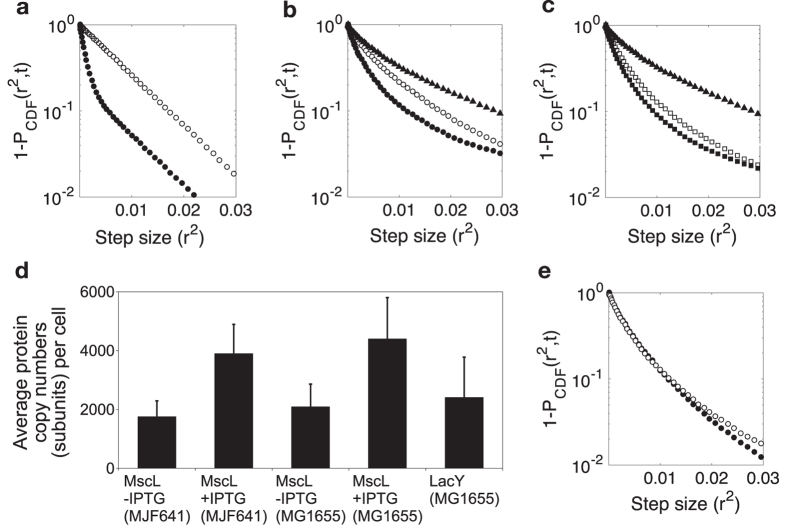
Cumulative distribution function (CDF) plotted as 1-P_CDF_ (**a**) of a simulated membrane protein with either a single population (open circles) or two populations (closed circles) with D_1_ = 0.06 μm^2^/s and D_2_ = 0.008 μm^2^/s and a hypothetical ratio of D_1_/D_2_ = 1:5. (**b**) CDF of MscL-mEos3.2 expressed in *E. coli* MG1655 with 0.1 mM IPTG (closed circles) and without IPTG (open circles). (**c**) CDF of MscL-mEos3.2 in *E. coli* MJF641 with (closed squares) and without (open squares) IPTG. In panel b and c the monomeric membrane protein fusion LacY-mEos3.2 expressed in MG1655 with 0.5% (w/v) L-rhamnose (triangles) was plotted as a control. (**d**) Average protein copy numbers per cell for the samples shown in panels b and c determined by qPALM. Numbers represent fluorescent subunits rather than fully assembled channels, and the error bars indicate the standard deviation of ~15 cells used for quantification. (**e**) CDF of MscS-mEos3.2 expressed in *E. coli* MG1655 with (closed circles) and without (open circles) IPTG.

The degree of heterogeneity of MscL-mEos3.2 super-complexes in the membrane was investigated using a cumulative distribution function (CDF). For individual step sizes within a fixed time interval of 31 ms, obtained from single-particle tracking, the CDF was plotted as 1-P_CDF_(r^2^,t) *versus* r^2^. As described in Oswald *et al*. the CDF of a homogeneously diffusing protein is characterized by exponential decay^30^, as simulated in Fig. 3a for a single population of freely diffusing membrane proteins (note the logarithmic y-axis). Two hypothetical populations at a ratio of 1:5 and diffusing with *D*_*1*_ = 0.06 μm^2^/s and *D*_*2*_ = 0.008 μm^2^/s show a kink in the distribution. The decay of the CDF of channels in *E. coli* MG1655 (IPTG induction) with mixed subunits of wild type and mEos3.2-tagged MscL is not simply biphasic (Fig. 3b), suggesting that multiple populations with different mobilities are present. In contrast, in non-induced *E. coli* MG1655 MscL diffuses more homogeneously.

In *E. coli* strain MJF641, in which all MscL subunits have mEos3.2 attached, the CDF shows behaviour similar to the induced state in MG1655, independent of whether the expression of the complexes was induced with IPTG or not (Fig. 3c). No significant difference in MscL abundance was observed between *E. coli* MJF641 and MG1655 as host (Fig. 3d). As a control for diffusion of a monomeric membrane protein, we expressed LacY-mEos3.2 from the *rha* promoter [0.5% (w/v) L-rhamnose] at levels similar to those of IPTG-induced MscL-mEos3.2. We plotted the CDF for LacY-mEos3.2 in Fig. 3b,c, which shows no visible cluster formation when expressed at similar levels as MscL-mEos3.2 (Fig. 3d). The diffusion of LacY-mEos3.2 is somewhat faster than that of MscL-mEos3.2 (Table 1), but the shape of the CDF of LacY-mEos3.2 is comparable to that of MscL-mEos3.2 in MG1655 (under non-inducing conditions; Fig. 3b), indicating that both are organized as monomeric membrane proteins. The deviation from a single exponential is likely caused by the fact that 3-dimentional diffusion trajectories are projected in two dimensions (on the focal plane), which distorts the distribution of step sizes^30^. Moreover, transient (weak) interactions between the proteins and components in the crowded membrane will cause anomalous diffusion, which is reflected in the CDF as well^31^. The MscS-mEos3.2 diffusion was independent from inducer concentrations in *E. coli* MG1655 (Fig. 3e) and the shape of the CDF indicates no cluster formation.

### Channel copy number and confinement

**Figure 4:**
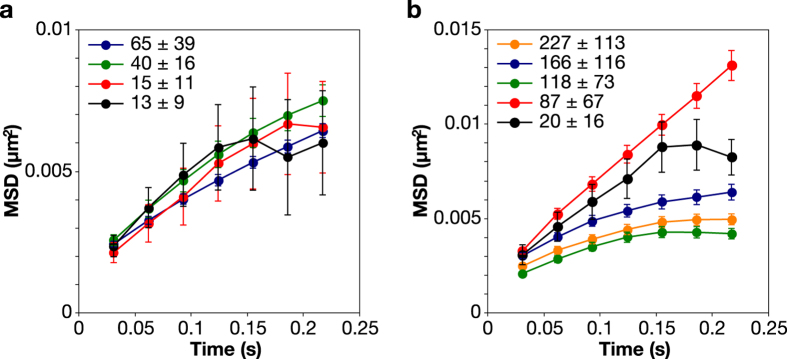
MSD plot of trajectories of mechanosensitive channels expressed in *E. coli* MJF641 (**a**) MscS-mEos3.2 and (**b**) MscL-mEos3.2 expressed from the *ara* promoter in pBAD. The numbers represent the average channel number per cell ± standard deviation, obtained from independent experiments by varying the inducer concentration from 0 to 0.5% L-arabinose. 200–2000 trajectories were analysed and error bars represent standard error of the mean (SEM).

To ascertain whether the self-association of MscL is a consequence of channel abundance and reflects the presence of the mEos3.2 fluorophore, we designed pBAD vectors to express the channels from the *ara* promoter (L-arabinose inducible), which is less leaky than the *trc* promoter. Hence, we could vary the expression levels over a much wider range, i.e. from a few to 65 (MscS) or several hundreds (MscL) of channels per cell. We also analysed the MSD of MscS and MscL in *E. coli* MJF641 when induced from pBAD (Fig. 4a,b, respectively), using a range of L-arabinose concentrations (0–1%). The mobility of MscS in MJF641 was independent of channel abundance but somewhat slower than in *E. coli* MG1655, which is possibly due to the larger drag on the channel because more fluorescent proteins are attached. The slope of the MSD of MscL decreased with increasing channel number per cell, which is indicative of clustering. If we assume a distribution of cluster sizes then the immobile fraction becomes more prominent as channel numbers increase. These data also suggest that mEos3.2 predisposes the MscL channel to form clusters. This may arise through collisions of pre-formed complexes rather than at the folding or assembly stage as fluorescent ß-barrel proteins typically mature (fold) more slowly than other proteins. In contrast, for MscS-mEos3.2 the spatial separation of the seven mEos3.2 fluorophores is sufficiently great that they cannot interact to form clusters. In this context, it is possibly significant that MscS can tolerate additions to the carboxy-terminus (e.g. the alkaline phosphatase protein), and the 18 residues at the carboxy-terminus can be deleted without significantly impacting folding, assembly or function^20^.

### Survival *versus* channel number

**Figure 5:**
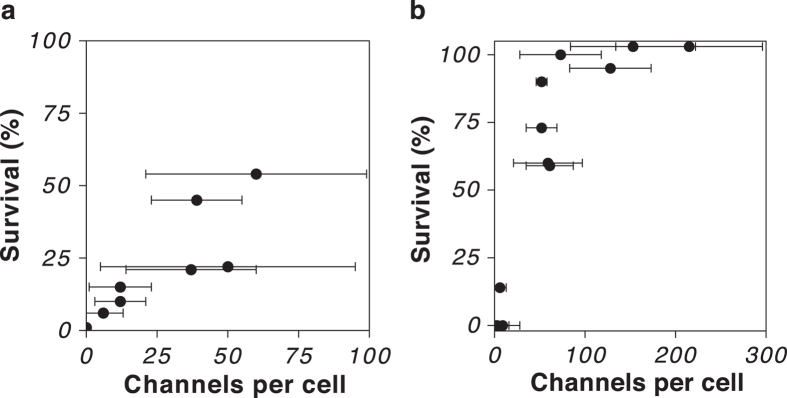
Survival after osmotic downshock of *E. coli* MJF641 as a function of fully assembled channels for (**a**) MscS-mEos3.2 or (**b**) MscL-mEos3.2. The osmotic downshock was applied by rapid mixing of cells adapted to LB plus 0.3 M NaCl into LB. The channel copy number was varied with different inducer concentrations (0 to 1% w/v L-arabinose). The average cell survival was determined by colony counting of shocked and non-shocked cells. The channels were quantified after cells were incubated for 5 h in PBS to ensure complete maturation of mEos3.2. For each condition between 6 and 25 cells were analysed by qPALM and the median of different assays with various inducer concentrations is plotted individually. Error bars represent standard deviation of quantified channel numbers.

An outstanding question is the number of channels that are required to confer protection from an osmotic downshock. We used *E. coli* MJF641 as the host strain and grew the cells in high-salt Luria Broth (LB containing 0.3 M NaCl; 0.78 ± 0.01 Osm), and varied the amounts of either MscS-mEos3.2 or MscL-mEos3.2 using the *ara* promoter system (L-arabinose at 0 to 1% w/v). MscK was not considered for this experiment, since its contribution to osmoprotection from a fast downshock is negligible. After the induction period of 1 h, a fast 0.3 M NaCl downshock was applied as described in Materials and Methods. At the same time the remaining non-shocked cells were washed twice in high-salt PBS (PBS adjusted to 0.78 ± 0.01 Osm with NaCl) and resuspended in equal volumes of the same buffer. Cells were kept in high-salt PBS for 5 h at 37 °C prior to quantification with qPALM to ensure complete maturation of all mEos3.2 proteins (maturation half time t_1/2_ = 40–45 min, see Supplementary Fig. S7). A detailed description of how channel numbers were quantified can be found in the Supplementary methods. The collective survivability-qPALM data is shown in Fig. 5a,b. For MscS and MscL channels, the survivability after the rapid 0.3 M NaCl downshock increased almost linearly with the number of channels per cell. Around 60% of the cells expressing MscS-mEos3.2 survived the downshock when 55 channels per cell were present. We could not increase the MscS channel number beyond this average, which may explain why a fraction of cells did not survive the osmotic downshock. With MscL-mEos3.2 we reached 100% survival with an average of 100 or more channels per cell.

To ascertain that *E. coli* is still viable and the MS channels are functional after the incubation of the cells in high-salt PBS, we adapted *E. coli* MJF641 to high-salt LB and expressed either MscS-mEos3.2 or MscL-mEos3.2 from the *ara* promoter [0.5% L-arabinose (w/v) for 1 h]. We then performed the osmotic downshock immediately after the induction period and after 5 h of incubation in high-salt PBS. Supplementary Fig. S6a shows that the overall number of cells only slightly decreased after 5 h in high-salt PBS, and the survival of cells before and after 5 h incubation in high-salt PBS was comparable (Supplementary Fig. S6b).

## Discussion

In bacteria MS channels play a key role in osmoregulation. MscS and MscL are the principal channels required for cells to survive hypoosmotic transitions. Other MS channels contribute at their usual levels of expression to varying extents but the ultimate outcome, cell survival, is dependent on complex parameters, including the rate of the shock^12^, specific channel gating properties, the overall number of MS channels and perhaps other as yet unidentified factors. Here, we provide a quantitative analysis of the contribution of MscS and MscL to osmoprotection and provide insights into their *in vivo* functioning. We determined channel abundance, aggregation state, localization in the membrane, and lateral diffusion. Despite the lack of organelles, it is increasingly clear that bacteria have well-organized compartments. Not only does the cytoplasm show confined structures, e.g. the nucleoid or separated areas of increased activity for transcription and translation^32^,^33^, but also the membrane seems to be more structured than initially expected for prokaryotic forms of life. The concept of bacterial lipid domains is under discussion, e.g. in *B. subtilis* processes such as peptidoglycan synthesis have been proposed to increase the local complexity of the cytoplasmic membrane^34^. The chemotaxis network in *E. coli* was shown to form clusters mainly at the cell poles, which might be a mechanism to increase their sensitivity by amplifying external signals from neighbouring receptors^4^,^35^. For mechanosensitive channels protein-lipid interactions form the basis for their gating and they are affected by membrane curvature. Thus knowledge about the localization and organization of the proteins in the membrane is crucial. We determined the localization of MscS, MscL and MscK in the *E. coli* plasma membrane with PALM; super-resolution optical microscopy has to the best of our knowledge not been used to localize MS channels. Reconstructions of cells expressing fluorescently labelled MscS, MscL and MscK showed an equal membrane staining. In a previous study, using FlAsH-tagged MscL and MscS, a small fraction of the channels localized to the poles of the cells^27^, however our results suggest that all channels tested have no preference for the cell poles.

The Saffman-Delbrück relationship for free diffusion in biological membranes predicts that the diffusion coefficient scales logarithmically with the hydrodynamic radius of the transmembrane domain^36^, which has been verified experimentally in proteoliposomes^37^. On the basis of the available crystal structures, the radii of the membrane-embedded domain of MscL and MscS are ~2.5 and 4 nm, respectively. There is no structural information of MscK but the protein is much larger (7 × 11 transmembrane segments, TMS) than MscS (7 × 3 TMS) and may have a radius of 7–8 nm. The here determined lateral diffusion coefficients suggest a stronger dependence on protein radius than predicted by the Saffman-Delbrück model. We note that the cytoplasm and periplasm are highly crowded and more viscous than water^38^,^39^. Since MscS has large cytoplasmic domains and MscK has large cytoplasmic and periplasmic domains, these proteins will experience more drag than MscL, which is not accounted for in the Saffman-Delbrück model. The retardation will be even more pronounced when one or more subunits are tagged with a fluorescent protein.

When MscL-mEos3.2 was expressed in *E. coli* MJF641, where each MscL subunit is tagged, channel clustering was observed even at the lowest levels of expression (Supplementary Fig. S4). This uneven distribution of channels is not what one would expect for Brownian diffusion of individual particles as shown by simulations (Supplementary Fig. S5). If only one or a few of the subunits of MscL were tagged with mEos3.2, the channels exhibited a high mobility and a more even distribution (Fig. 2). At higher levels of expression of tagged proteins, clusters were formed even in the presence of wild type polypeptides. We attribute the clustering of MscL as labelling artefacts that appear when the majority or all of the subunits of the pentameric channels are tagged with mEos3.2. Our single-particle tracking experiments are entirely consistent with the observation of clusters in PALM reconstructions. We find that the diffusion of MscL (clustered in *E. coli* MJF641) is anomalous with signs of confinement at longer timescales, especially with increasing channel copy numbers (Fig. 4b). The cumulative distribution function provided quantitative information on the cluster formation that cannot be deduced from the mean square displacement analysis. Where clustering was observed the CDF showed multiple populations. We were not able to fit the data with a multicomponent linear equation since the graph shows a smooth transition from slowly diffusing clusters to faster, smaller aggregates and individual channels. We used LacY-mEos3.2, a well-characterized, monomeric membrane protein, as a control and observed that the shape of the CDF of MscL-mEos3.2 (at low inducer concentration) and LacY-mEos3.2, expressed in *E. coli* MG1655 point towards a similar diffusional behaviour with no signs of protein clustering.

**Figure 6:**
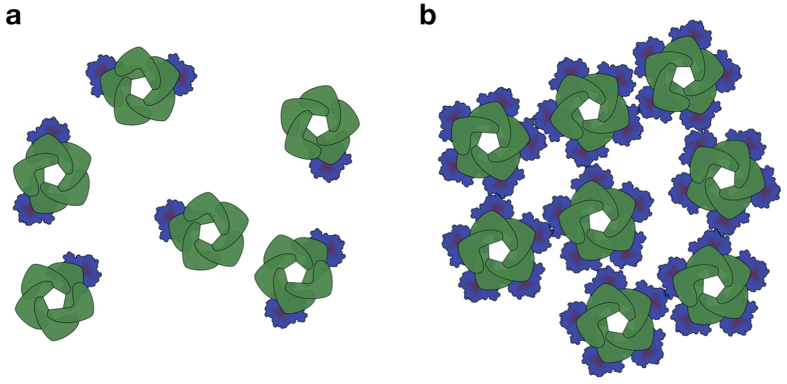
Cluster formation of MscL depends on the number of mEos3.2-labelled subunits in the channels. (**a**) In *E. coli* MG1655, using mild expression conditions, the plasmid-derived MscL-mEos3.2 mixes with chromosome-derived MscL subunits. In most cases only one or two subunits are labelled. (**b**) In *E. coli* MJF641, all subunits are labelled, which reduces rotational freedom of the fluorescent protein and leads to artificial cluster formation of MscL channels. Cluster formation can also occur when MscL-mEos3.2 is overexpressed in *E. coli* MG1655, that is, when the majority of subunits have a fluorescent protein moiety.

In theory, cluster formation can occur when channels have a minimum of two fluorescent tags weakly interacting with each other. Different scenarios of expressing MscL-mEos3.2 in *E. coli* MG1655 or MJF641, are depicted in Fig. 6a,b. Remarkably, under conditions where we find (predominantly) clusters, the channels protected *E. coli* against hypoosmotic stress and the conductance and gating properties were similar to those of (non-clustered) wild type MscL. In our studies, individual channels are tied together via the cytoplasmic mEos3.2 label and not by the tension-receiving transmembrane surface, which apparently does not affect the gating activity. It should be noted though that other studies have observed MscL clustering in *E. coli*^40^,^41^ as well. In these studies MscL was labelled with GFP, which has a much higher tendency for self-association than mEos3.2. Grage *et al*. observed clustering of an Alexa-488 labelled MscL reconstituted in lipid vesicles by small-angle neutron scattering (SANS), atomic force microscopy (AFM), and fluorescence microscopy^28^. Their continuum mechanics simulations shows that organizing MscL channels in clusters minimizes their energy and lowers their activity. In brief, they argue that channel opening increases their surface area and clustering of the proteins would thus hamper their gating. Thus clustering of MscL could be an activity regulation mechanism, e.g. by membrane-mediated (anti−) cooperative gating^42^,^43^, however our data indicate that native channels do not cluster.

We provide evidence that genetic labelling of a membrane protein like MscL can give rise to artefacts, in particular when proteins are overexpressed. Fluorescent proteins can cause mislocalization when fused to homooligomers^44^ and have the tendency to self-associate. Even though monomeric variants like mEos3.2 have been engineered with a *K*_*d*_ for self-association >480 μM^45^, when linked to a membrane protein artificial clustering may still occur. For membrane proteins one needs to take into account the net effect of local protein concentrations, orientational restriction, and volume exclusion^46^. The largest contribution to the enhancement of self-association of proteins in membranes originates from the restriction in translational mobility, which manifests itself as an increased local concentration. Grasberger *et al*. estimated that for a cylindrical-shaped membrane protein its propensity to form dimers can be 10^6^-times higher than for isotropic diffusion of water-soluble proteins^46^. This situation may not hold for MscL-mEos3.2, since the putative oligomer-forming moiety is not embedded in the membrane but rather connected to a membrane protein via a small linker. The rotational freedom of mEos3.2 is presumably higher than that of the membrane protein itself, but certainly lower compared to a cytoplasmic protein of the same size. The situation becomes more pronounced when five fluorescent moieties are situated in close proximity to each other and to the membrane, which is the case when all MscL subunits are labelled with mEos3.2 (Fig. 6b). We note that MscS and MscK, with seven subunits, did not show the clustering behaviour. From crystal structures it is known that MscL subunits are more closely packed to each other at the C-terminus than those of MscS. The rotational freedom of mEos3.2 in MscL may thus be much smaller than in MscS. This decrease in entropy may lead to dimerization of mEos3.2 not only within a channel but also between channels. The clustering of channels through mEos3.2 apparently leaves sufficient freedom for MscL to undergo the large conformational changes from closed to open.

Li and co-workers used a genome-wide approach, based on ribosome profiling, to determine protein copy numbers by assuming a constant rate of protein synthesis and turnover for all bacterial proteins^47^. Their deduced copy numbers for MscS and MscL suggest that they are relatively abundant in the cell (around 600 channels of MscS and MscL in cells growing in rich medium). Using Western blot analysis and fluorescence microscopy, MscL was detected in the range of 300–1000 channels per cell^40^. Electrophysiology studies showed lower copy numbers ranging from 20–40 MscS and 5–40 MscL channels per cell^48^,^49^, which most likely reflects the fact that not all channels gate in patch clamp experiments, whereas Western blotting or ribosome profiling detects the entire amount. With the multiplicity of MS channels in *E. coli* and deduced protein numbers, the question arose, how many channels are required to confer protection from a given downshock.

To address the relationship between cell survival after hypoosmotic shock and channel abundance, we performed viability experiments based on colony counting, using an *E. coli* strain lacking all seven native MS channels (MJF641) and expressing various amounts of plasmid-derived MscS-mEos3.2 or MscL-mEos3.2. We observe a variation in survivability of 15% or higher, which is not unusual for colony counting in plating experiments^50^. Alternatively, survivability can be determined with flow cytometry^51^ or by time-lapse imaging in microfluidic flow cells^12^. We developed a reliable protocol of protein quantification by qPALM, which allowed us to correlate the cell survival with channel copy numbers for each expression condition. Our qPALM approach is based on the work of Annibale *et al*.^52^ but tailored for *in vivo* application with diffusing fluorescent probes. Quantitative microscopy faces several challenges, which can lead to over- or underestimation of the protein copy numbers. Detailed knowledge about the photo-physical properties of the fluorescent protein allows correcting for photo-blinking and maturation. However, the photo efficiency of photoactivatable proteins poses some uncertainty that we could not correct for. The determined copy numbers therefore represent a lower boundary and might be somewhat higher (see Supplementary methods and Supplementary Figs S7–S9 for more information).

We found that the channel numbers correlate more or less linearly with survival after a 0.3 M NaCl downshock. Approximately 100 MscL-mEos3.2 channels are required for full protection (see Fig. 5). We did not succeed in expressing MscS-mEos3.2 to similarly high levels and thus did not reach full protection, but by extrapolation the same number may apply for MscS. The highest MscL channel number we achieved by induction with L-arabinose was on average 400 per cell, which is close to native expression based on ribose profiling and immunoblotting^40^,^47^. It was recently shown that the speed at which the cells experience the downshock is an important determinant for cell survival^12^. The chances of cell survival significantly increased if the downshock was applied over a longer period, and with slow enough downshock even MscK protected the cell from lysis. Thus, our correlation between cell survival and channel numbers shown in Fig. 5 is valid for a fast 0.3 M NaCl downshock applied by rapid pipetting. Booth calculated that upon a 0.5 M downshock five MscS channels would in theory suffice to deplete the cell of its ion pool within 200 ms^53^. Our results suggest that the flux through the channel is lower or that gating takes longer than is typically assumed. One reason might be that during an osmotic downshock the membrane potential collapses and consequently the flux of solutes through the channels is substantially reduced.

In summary, we show for MscL that above 100 channels all cells survive the imposed osmotic stress and that a similar quantitative relationship is likely for MscS. Additional channels might be needed when cells face a downshock of higher magnitude^19^. We show that MscS, MscL and MscK are homogenously distributed over the membrane with no indications for clustering of the channels themselves. The diffusional behaviour of MscL and MscS is that of free channels with some indications for confinement on longer timescales, which may reflect heterogeneity in macromolecular crowding or local differences in lipid composition of the membrane.

## Materials and Methods

### Electrophysiology

Patch clamp recordings were conducted on membrane patches derived from giant protoplasts using the strain MJF429 (Δ*MscS*, Δ*MscK*) transformed with pTRC-MscS or pBAD-MscS-mEos3.2 and MJF453 (Δ*MscL*, Δ*MscK*) with pBAD-MscL-mEos3.2 plasmids. The cultures were induced with 1mM IPTG for 15 min (pTRC-MscS) or 0.5% L-arabinose for 65 min (pBAD-MscS-mEos3.2 and pBAD-MscL-mEos3.2) before protoplast formation. Excised, inside-out patches were analysed at membrane potential of −20 mV with pipette and bath solutions containing 200 mM KCl, 90 mM MgCl_2_, 10 mM CaCl_2_, and 5 mM HEPES buffer at pH 7.0. All data were acquired at a sampling rate of 50 kHz with 5-kHz filtration using an AxoPatch 200B amplifier and pClamp software (Molecular Devices). The pressure threshold for activation is shown as the pressure ratio between MscL and MscS (P_L_∶P_S_).

### Osmotic downshock assay

*E. coli* MJF641 harbouring pTRC-MscS-mEos3.2 or pTRC-MscL-mEos3.2 was grown overnight at 37 °C in LB + 50 μg/mL ampicillin. The next day cultures were diluted to OD_650_ = 0.05 in 10 mL pre-warmed LB and incubated until OD_650_ = 0.4. Cultures were 10x diluted into a high-salt medium (LB + 0.3 M NaCl) and 0.3 mM IPTG was added when OD_650_ reached ~0.2. At OD_650_ = 0.3 an osmotic downshock was applied by diluting the cells 1:20 into LB medium (downshock) or into LB medium + 0.3 M NaCl (control). After 10 min of incubation at 37 °C serial dilutions were made (10^−1^ to 10^−5^) and 5 μL of each dilution was spotted in quadruplicate onto LB-agar plates (shock) or LB-agar plates containing additional 0.3 M NaCl salt (control). Survival was determined by comparing colony forming units on shock and control agar plates after overnight incubation at 37 °C.

### Survival *versus* channel number

Cultures of *E. coli* MJF641 harbouring pBAD-MscS-mEos3.2 or pBAD-MscL-mEos3.2 were grown overnight in LB plus 100 μg/mL ampicillin at 37 °C. The next morning a 1:100 dilution was made in 10 mL pre-warmed LB. When an OD_650_ = 0.4 was reached cultures were diluted 1:40 into 20 mL LB + 0.3 M NaCl to adapt to high salt, and the culture incubated until OD_650_ = 0.08. At this point freshly prepared L-arabinose was added at final concentrations of 0.01%, 0.1%, 0.25%, 0.5% and 1% (w/v). After 1 h induction (optical density ~0.3–0.4), an osmotic downshock was applied by rapidly pipetting 0.5 mL of the cells into 10 mL LB (shock) and 10 mL LB + 0.3 M NaCl (control). Cells were incubated for 10 min at 37 °C prior to serial dilution and plating onto LB-agar or LB-agar + 0.3 M NaCl plates (described above). The survival of uninduced cells was also assessed. Concomitantly with the shock step, the remaining culture of cells grown in LB + 0.3 M NaCl was collected by centrifugation (~15 mL) for qPALM or filtration (for future downshock) and washed twice in PBS high salt (PBS adjusted with NaCl to the same osmolarity as LB to avoid downshock). Cells in PBS high salt were incubated at 37 °C for 5 h prior channel quantification with qPALM, to complete maturation of mEos3.2. At this point cells were also tested for survivability by dilution into PBS (shock) or high-salt PBS (control), after 10 minutes they were serially diluted in the same buffer and plated onto LB-agar or LB-agar + 0.3 M NaCl plates.

### Sample preparation for microscopy

Coverslips (Carl Roth, LH26.1) were cleaned with 5 M KOH in a sonication bath, plasma-cleaned for 10 min and coated with 2% (v/v) (3-Aminopropyl)trioxysilane (Aldrich) in acetone for 15 min. Cells were grown as described in Supplementary methods and prepared for image acquisition in exponential growth phase (OD_600_ of 0.2–0.4). Cells were immobilized on coated coverslip with a second cleaned coverslip on top to prevent evaporation.

### PALM setup

For single molecule imaging a home-built inverted microscope based on an Olympus IX-81 microscope with a high numerical aperture objective (100 X, NA = 1.49, oil immersion, Olympus, UApo) was used. Solid-state lasers were from Coherent (Santa Clara, USA): 405 nm (Cube, 100 mW) and 561 nm (Sapphire 561, 100 mW). Laser beams were collimated with lenses and combined using dichroic mirrors. Laser power for mEos3.2 readout (561 nm) was set to ~1 kW/cm^2^. The power for mEos3.2 activation (405 nm) was adjusted for each experiment based on labelling density. Imaging was performed in semi-TIRF and fluorescence was recorded using an electron multiplying charge coupled device (EM-CCD camera) from Hamamatsu, Japan, model C9100-13.

### PALM data acquisition and analysis

To activate mEos3.2 molecules a pulsed laser was used for illumination. To detect single molecules typically 150–250 cycles of photoactivation were repeated with each cycle consisting of 20 frames. The first frame was used to switch mEos3.2 from a green to a red state, using a UV laser (405 nm). The following 19 frames were used to readout the activated fluorescent proteins in the red channel (561 nm). Usually the read-out laser photobleaches all activated mEos3.2 molecules during these 19 frames so that the next cycle of photoactivation can be started. The exposure time of each frame was set to 31 ms. For qPALM up to 800 cycles were recorded to ensure that every mEos3.2 molecule was activated during the time of image acquisition. All measurements were carried out at 22 °C. The acquired movies were analysed with a home-written ImageJ plugin. In the reconstructed images each fluorescent molecule was represented as a single spot at its determined coordinates with a brightness that corresponds to the localization accuracy.

### Single-particle tracking

The PALM data from MS channel fusions with mEos3.2 can be used to determine the diffusion constant *D*. A home-written ImageJ plugin was used to detect two fluorescent proteins in two consecutive frames that are in close proximity to each other (up to 320 nm), which resulted in trajectories of various lengths. The diffusion constant was calculated from the mean square displacement (MSD) by averaging ~10,000 trajectories that had a minimal length of 5 frames. The MSD curves were linearly fitted for points in the range of 0.03 to 0.25 s and diffusion coefficient *D* was calculated assuming 2D diffusion.

### qPALM

We developed a protocol for determining the channel numbers in live *E. coli* cells that allows for accurate quantification even at low levels of expression. To obtain reliable copy numbers we included (i) the maturation time, (ii) a correction for over counting due to grouped traces, (iii) blinking behaviour of the fluorescent protein, and (iv) a correction for the fact that only a fraction of the cell are analysed, because of the limiting depth of field. A detailed description is given in Supplementary methods.

## Additional Information

**How to cite this article**: van den Berg, J. *et al*. On the mobility, membrane location and functionality of mechanosensitive channels in *Escherichia coli*. *Sci. Rep.* **6**, 32709; doi: 10.1038/srep32709 (2016).

## Supplementary Material

Supplementary Video S1

Supplementary Video S2

Supplementary Information

## Figures and Tables

**Table 1:** Diffusion coefficients *D* for MscS-mEos3.2, MscL-mEos3.2 and MscK-mEos3.2 channels in *E. coli* MG1655, using leaky expression (−IPTG) and with 0.1 mM inducer (+IPTG).

Condition	MscS −*IPTG*	MscL −*IPTG*	MscK −*IPTG*	MscS +*IPTG*	MscL +*IPTG*	MscK +*IPTG*	LacY
D (*10^−2^ μm^2^/s)	2.81	5.80	2.09	2.67	0.62	1.72	8.10

LacY-mEos3.2 was induced from the *rha* promoter in pACYC.
