# Supplementary figures and images for: On the mobility, membrane location and functionality of mechanosensitive channels in Escherichia coli
Source: Sci Rep. 2016 Sep 6;6:32709. doi: 10.1038/srep32709 (PMC5011748; doi:10.1038/srep32709)

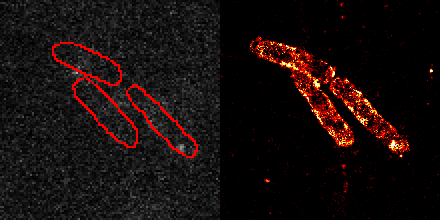

Supplement: Supplementary Video S1 [file srep32709-s1.gif]

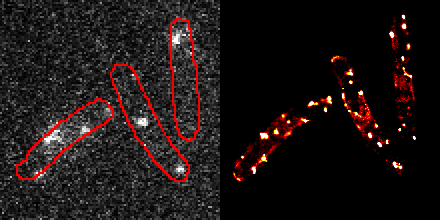

Supplement: Supplementary Video S2 [file srep32709-s2.gif]
