# Supplementary material for: On the mobility, membrane location and functionality of mechanosensitive channels in Escherichia coli
Source: Sci Rep. 2016 Sep 6;6:32709. doi: 10.1038/srep32709 (PMC5011748; doi:10.1038/srep32709)
Supplement: Supplementary Information [file srep32709-s3.pdf]

Supplementary information

On the mobility, membrane location and functionality of mechanosensitive channels in *Escherichia coli*

Jonas van den Berg[1], Heloisa Galbiati[2], Akiko Rasmussen[2], Samantha Miller[2] and Bert Poolman[1]

[1]Department of Biochemistry, Groningen Biomolecular Sciences and Biotechnology & Zernike Institute for Advanced Materials, University of Groningen, 9747 AG Groningen, The Netherlands

[2]School of Medicine, Medical Sciences and Nutrition, Institute of Medical Sciences, University of Aberdeen, Foresterhill, Aberdeen AB25 2ZD United Kingdom

To whom correspondence should be addressed: b.poolman@rug.nl

Supplementary Methods

Supplementary Tables: Table S1-S3

Supplementary Figures: Fig. S1-S9

Supplementary Video legends: S1-S2

SUPPLEMENTARY METHODS

Strains and plasmids. *E. coli* strains MG1655 and MJF641 (Δ7) were used for our studies. Patch clamp recordings were done in *E. coli* MJF429 and MJF453 and maturation analysis in *E. coli* MC1061. All strains are described in Table S1. To create the channel fusions in pTRC, all pTRC-MscX vectors (X refers to any of the MS channel proteins) were linearized by *Xho*I digestion. The unique *Xho*I site is at the end of each *mscX* gene and immediately before the DNA encoding the poly-His tag. mEos3.2 was amplified from the pBAD-mEos3.2 plasmid by Polymerase Chain reaction (PCR). Primers to amplify mEos3.2 had a 15 bp sequence homologous to both extremes of the linearized pTRC-MscX, allowing the ligation of the mEos3.2 PCR products into the pTRC sequence. The ligation step was performed using the CloneEZ® PCR Cloning Kit (GenScript). For cloning the fusion constructs in pBAD the USER™ enzyme (Uracil-Specific Excision Reagent Enzyme) was used. In this approach primers are designed to have a selected T nucleotide replaced by a U (see Table S3). Amplified sequences included the backbone (pBAD), genes (*mscS*, *mscL* and *mscK*) and the fluorescent protein (*mEos3.2*). All plasmids and primers are listed Table S2 and Table S3.

Growth conditions. Transformed cells were grown overnight either in EZ rich defined medium (Teknova) supplemented with 0.2% (w/v) glycerol or Luria-Bertani (LB) medium in the presence of 50 or 100 µg/mL ampicillin. Cultures were incubated at 37 ºC under continuous shaking at 200 rpm and measured in exponential growth phase (OD_{600} ~ 0.3-0.5) unless otherwise stated.

Quantification of MS channels fused to mEos3.2 using qPALM. Quantitative photo-activated localization microscopy (qPALM) of fluorescently labelled membrane proteins requires detailed knowledge about the photophysics of the fluorescent protein. The fluorescent protein mEos3.2 was chosen, because of its superior properties in term of brightness and monomeric character[1]. Photo switching was well controlled by varying the power of the activating laser (405 nm), making it ideal for quantitative microscopy. We developed a protocol for determining the channel numbers in live *E. coli* cells that allows quantification even under low

expression conditions. To obtain reliable copy numbers we included in the analysis: (i) the maturation time, (ii) a correction for over counting due to grouped traces, (iii) blinking behavior of the fluorescent protein, and (iv) a correction for the fact that only a fraction of the cell can be seen, because the depth of field is limited.

(i) Maturation time. We determined the *in vivo* maturation time of the fluorescent protein by expressing soluble mEos3.2 protein in *E. coli* MC1061 under anaerobic growth conditions at 37 °C. After addition of 32 µg/mL chloramphenicol the culture was aerated and the emerging fluorescence was measured using flow cytometry, as shown in Fig. S7. We observed maturation of mEos3.2 with two distinct kinetic phases. The maturation half times ($t_{1/2}$) were obtained by fitting the data points to a multi-exponential function. We find $t_{1/2}$ values for mEos3.2 maturation in live *E. coli* cells at 37 °C of <5 min for the first phase and 40-50 min for the second phase. Cells used for channel counting were kept for 5 h at isosmotic PBS at 37 °C in all cases to assure full maturation (avoiding undercounting due to incomplete maturation).

(ii) Single molecules as grouped fluorescent traces. One convenient fact when quantifying membrane proteins is their slow diffusion. Membrane proteins hardly change their position in between two frames. Proteins that are fluorescent for several consecutive frames can therefore be grouped together and counted as one molecule. We immobilized purified mEos3.2 on a clean cover slide and observed its fluorescent properties using the same laser settings as for a live sample. The counts obtained from PALM were scanned for grouped traces using a custom written ImageJ macro. The algorithm finds traces by grouping counts that occur within a radius of 4 pixels for a series of frames. The distribution of the fluorescence ON time (t_{on}) was plotted as number of frames in Fig. S8a. Counts that were only detected in one frame and cannot be grouped are represented in magenta. Traces longer than one frame can be fitted with an exponential y = a*exp(b*x); the obtained values were b = -0.06778 and a = 29,128. The resulting fit reflects the t_{on} of long-lived molecules very well, but the measured counts for short-lived molecules with a t_{on} of 1 frame or less are higher than predicted from fitting and extrapolating to $x = 1$. We assume that the t_{on} of fluorescent proteins follows a single exponential decrease and determine the number of mEos3.2 molecules as defined by the theoretical, extrapolated fit (blue bar Fig. S8a). The counts represented by the magenta bar can be attributed to background and auto

fluorescence, which is wrongly detected by the algorithm. Like that the half-time of the fluorescence $t_{on\ 1/2}$ of mEos3.2 was calculated to be 30.7 ms. For all live samples we used the same fit to estimate the number of ungrouped molecules. Fig. S8c-e show the distribution of ON times for three expression levels induced with 1%, 0.25% and 0.01% in descending order. Finally, it is essential that all fluorescent proteins present in the sample are activated within the time of acquisition. The acquisition needs to last at least until the time when the total detected counts per frame reach zero, as can be seen in Fig. S8b.

(iii) Blinking. mEos3.2 can exist in three different states: activated, dark and bleached. Many fluorescent proteins can blink, which means they can repeatedly switch between activated and dark states, before they bleach. This leads to overestimation of protein copy numbers when grouping fluorescent traces. We corrected for that by introducing a dark time t_d in our analysis, in which the protein is dark, but not yet bleached and can reoccur after several frames. This additional parameter must be chosen carefully when grouping fluorescent traces because it defines how long a fluorescent protein is allowed to stay in a dark state in order to be counted as one grouped trace. When the dark time t_d is not long enough one fluorescent protein might be interpreted as more than one molecule. In the work of Annibale et al. the photoblinking behaviour of mEos2 was analysed[2]. We adapted their strategy and tailored it for our purpose. They observed that with longer t_d the counts of immobilized mEos2 molecules reach a more accurate number. They found that the decreasing curve could be fitted with the following semi-empirical equation:

$$N(d_t) = N \cdot \left(1 + n_{blink} \cdot e^{\frac{1-d_t}{t_{off}}}\right)$$

where N represents the actual number of fluorescent proteins in the sample, n_{blink} is the number of blinks and t_{off} the dark time. However, there is a downside when quantifying proteins in live cells, which has to do with their diffusion during the image acquisition. This makes qPALM on live cells challenging, because even membrane proteins can diffuse long distances during image recording of several minutes. In the previous section the method for determining grouped traces is described. To correct for the fact that the detected counts do not allow any dark time (t_d = 0), we determined the blinking behaviour of mEos3.2 immobilized on cover slides as described before. In Fig. S9 we plot the counts per µm^2 as a function of dark

time t_d. The ratio of counts obtained from fitting the curve with the semi empirical equation and the counts detected without dark time is $\gamma = \frac{N}{N_{gt}} = 0.697$, which is the correction factor for fluorophore blinking. Using this approach we reduce the risk of overcounting due to blinking. It should be mentioned that this attempt fails to give accurate numbers when not all mEos3.2 proteins are activated. In Ulrich et al. they find about 20% of GFP-type fluorescent proteins stay in the dark and will thus not be detected[3]. Because photo efficiency is dependent on many factors, like pre-treatment of the sample, type of organism and laser intensity for activation and readout, we decided not to correct for photo efficiency. We likely underestimate the copy numbers for MS channels by about 20%. The actual channel numbers will thus be somewhat higher depending on the photo efficiency of mEos3.2.

(iv) Depth of field. We determined the depth of field to estimate what fraction of the cell is out of the observable area. We performed a z-stack with epi-illumination over a range of 4 µm with increments of 0.02 µm on immobilized fluorescent beads and determined the upper and lower limit at which the peak fitter can still detect the fluorophores. The laser power was decreased to match the intensity profile of the fluorescent beads to the one from mEos3.2 molecules. The depth of field was determined to be $d_z = 0.78$ µm. With a width of 1 µm an *E. coli* cell would almost fit completely in the depth of field, except the upper and lower part of the cell's cylinder including the membrane. Due to the relatively big depth of field, we decided not to introduce a correction for the limiting depth of field, because proteins are very likely to diffuse to the observable area of the membrane within the long acquisition time.

SUPPLEMENTARY TABLES

Table S1. *E. coli* strains used in this study

E. coli strain	Genotype	Reference
MJF641	Frag1, Δ*kefA(mscK)::*kan, Δ*yjeP*, Δ*yggB*(*mscS*), Δ*F786*(*ybiO*), *mscL*::cm, *ybdG*::Apr, F343⁻(*ynaI*), *ycjM*::Tn10)	[4]
MG1655	K-12, F⁻λ⁻*ilvG-rfb*-50 *rph*-1	[5]
MJF429	Frag1, Δ*yggB(mscS)*, Δ*kefA(mscK)*::*kan*	[6]
MJF453	Frag1, Δ*kefA(mscK)*::*kan*, Δ*mscL*::*Cm*	[6]
MC1061	K-12, F⁻ λ⁻ Δ*(ara-leu)7697* [*araD139*]B/r Δ*(codB-lacI)3 galK16 galE15* e14⁻ *mcrA0 relA1 rpsL150*(Str^R) *spoT1 mcrB1 hsdR2*(r^-m^+)	[7]

Table S2. Plasmids used in this study

Plasmid	Description	Reference
pTRC-MscS	*Nco*I-*Hind*III *mscS*-bearing fragment cloned into *Nco*I-*Hind*III sites of pTRC99a plasmid backbone	[8]
pTRC-MscL	*Nco*I-*Hind*III *mscL*-bearing fragment cloned into the pTRC-MscS backbone	This study
pTRC-MscK	*Nco*I-*Hind*III *mscK*-bearing fragment cloned into *Nco*I-*Hind*III sites of pTRC99a plasmid backbone	Kind gift from T. Rasmussen
pTRC-MscS-mEos.3.2	mEos3.2 fragment with 15 bp homology to both extremes of the linearized pTRC-MscS cut by *Xho*I. The cloning tagged mEos3.2 at the C-terminal sequence of MscS	This study
pTRC-MscL- mEos3.2	mEos3.2 fragment with 15 bp homology to both extremes of the linearized pTRC-MscL cut by *Xho*I. The cloning tagged mEos3.2 at the C-terminal sequence of MscL	This study
pTRC-MscK-mEos3.2	mEos3.2 fragment with 15 bp homology to both extremes of the linearized pTRC-MscK cut by *Xho*I. The cloning tagged mEos3.2 at the C-terminal sequence of MscK	This study
pBAD-mEos3.2	*Nco*I-*Xma*I *mEos3.2* fragment cloned into *Nco*I-*Xma*I sites of pBAD plasmid backbone	Kind gift from A. Robinson
pBAD-cLIC-GFP	*Swa*I–*Xba*I fragment of pBAD-cLIC and an *Xba*I-digested PCR product holding an *Nco*I-free sequence coding for EGFP	[9]
pBAD-MscS-mEos3.2	*mscS* and mEos3.2 genes with 15 bp homology to each other and to the pBAD vector were cloned with a linker (GGENLYFQ) separating *mscS* and mEos3.2	This study
pBAD-MscL-mEos3.2	*mscL* and mEos3.2 genes with 15 bp homology to each other and to the pBAD vector were cloned with a linker (GGENLYFQ) separating *mscL* and mEos3.2	This study
pACYC-LacY-mEos3.2	*Nco*I and *Bam*HI LacY-mEos3.2 fragment cloned into pACYC backbone	This study

Table S3. Primers used in this study

**Primers**	**Template for PCR**	**Region amplified**	**Primer sequence (5' to 3')**
mEos3.2 Fwd[a]	pBAD-mEos3.2	mEos3.2	gaagacaaagctgcgatgggaagtgcgatt
mscS Fwd	pTRC-MscS	mscS	attaacca**u**ggaagatttgaatgttgtcgatagc
mscL Fwd	pTRC-MscL	mscL	attaacca**u**gagcattattaaagaatttcgcgaatttgc
mscK Fwd	pTRC-MscK	mscK	attaacca**u**gactatgttccagtattacaaacgatc
mEos3.2 Fwd[b]	pBAD-mEos3.2	mEos3.2	aggggaaaa**u**ttatattttcaaggtggaagtgcgattaagccag
pBAD Fwd	pBADcLIC_GFP	pBAD His tag for insertion of genes	accaccacca**u**catcatcatcaccatcattaagtcg
mEos3.2 Rev[a]	pBAD-mEos3.2	mEos3.2	gtggtggtggtggtggccctggaagtacag
mscS Rev	pTRC-MscS	mscS	attttcccc**u**cccgcagctttgtcttctttcac
mscL Rev	pTRC-MscL	mscL	attttcccc**u**ccagagcggttattctgctctttc
mscK Rev	pTRC-MscK	mscK	attttcccc**u**cctgcggccgcaagctttacg
mEos3.2 Rev[b]	pBAD-mEos3.2	mEos3.2	atggtggtgg**u**gtcgtctggcattgtcagg
pBAD Rev	pBAD-cLIC_GFP	pBAD	atggttaa**u**tcctcctgttagcccaaaaaac

[a]For amplification of mEos3.2 for pTRC-MscX-mEos3.2
[b]For amplification of mEos3.2 for pBAD-MscX-mEos3.2

SUPPLEMENTARY FIGURES

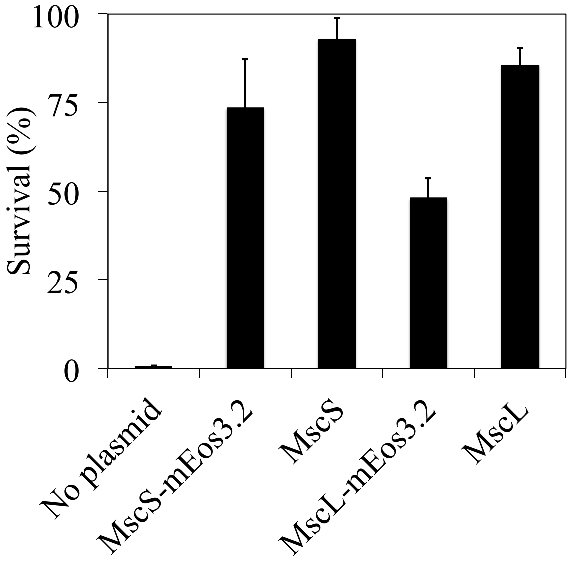

Fig. S1. Survival of *E. coli* MJF641 cells transformed with MscS or MscL with and without mEos3.2 tag. Samples were induced with 0.3 mM IPTG for 15 min prior to being subjected to a 0.3 M NaCl downshock. As a control *E. coli* MJF641 without plasmid was subjected to the same osmotic downshock. Error bars represent standard deviation.

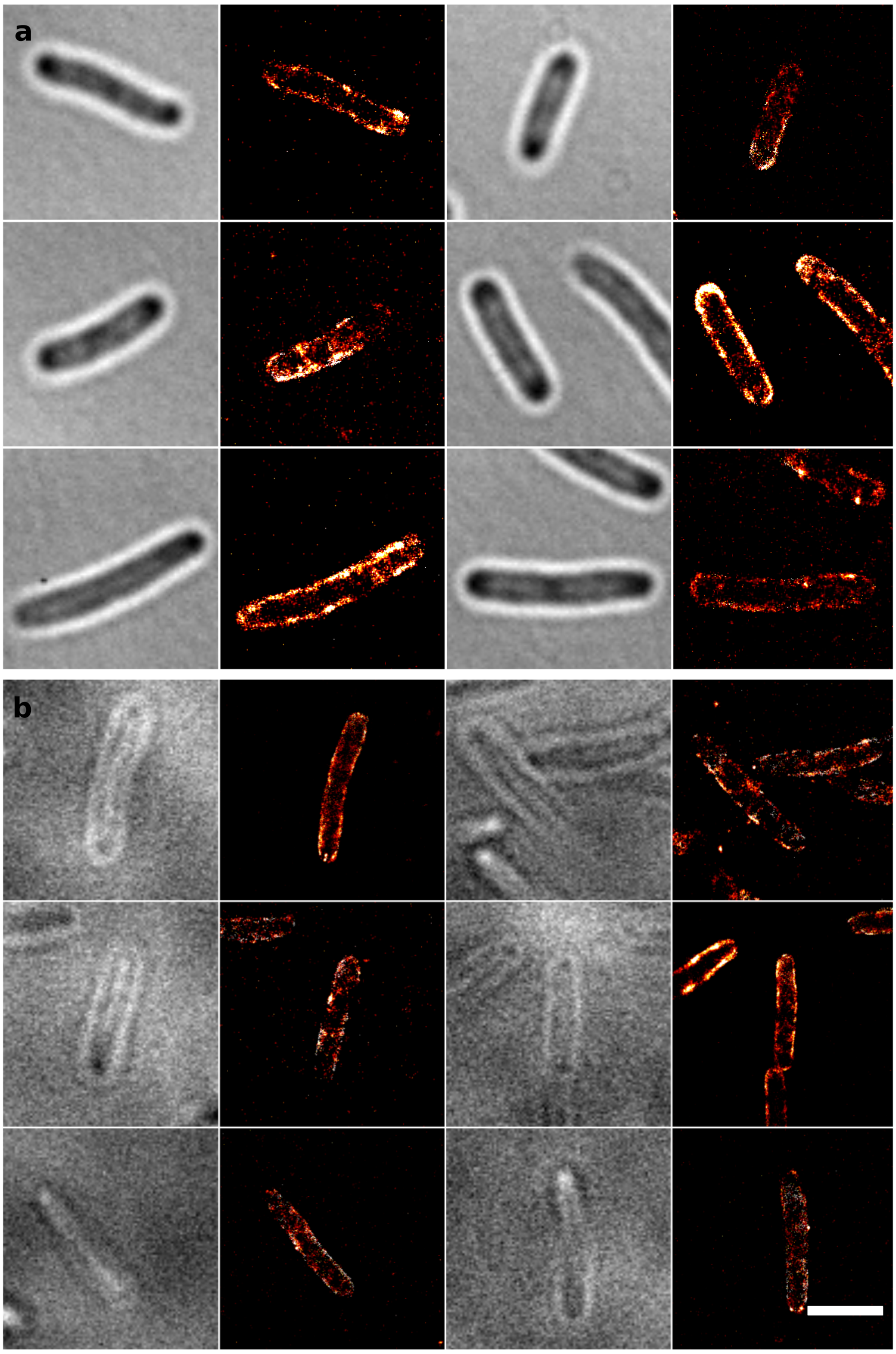

Fig. S2. PALM reconstructions of *E. coli* MG1655 harbouring pTRC-MscS-mEos3.2 with (a) leaky expression and (b) induction with 0.1 mM IPTG for 30 min. Scale bar: 2μm.

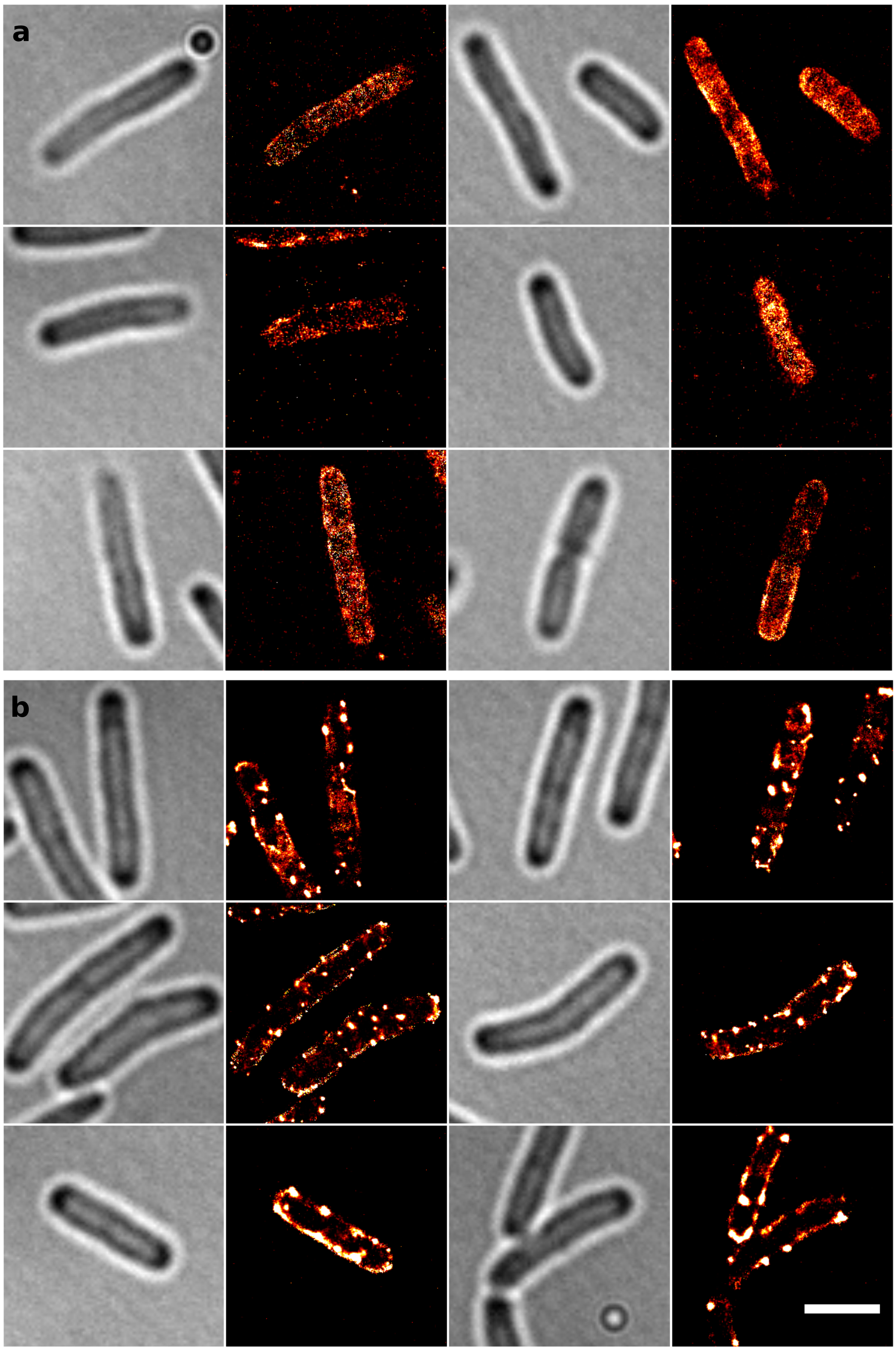

Fig. S3. PALM reconstructions of *E. coli* MG1655 harbouring pTRC-MscL-mEos3.2 with (a) leaky expression and (b) with 0.1 mM IPTG for 30 min. Cluster formation is visible in cells that were induced. Scale bar: 2 µm.

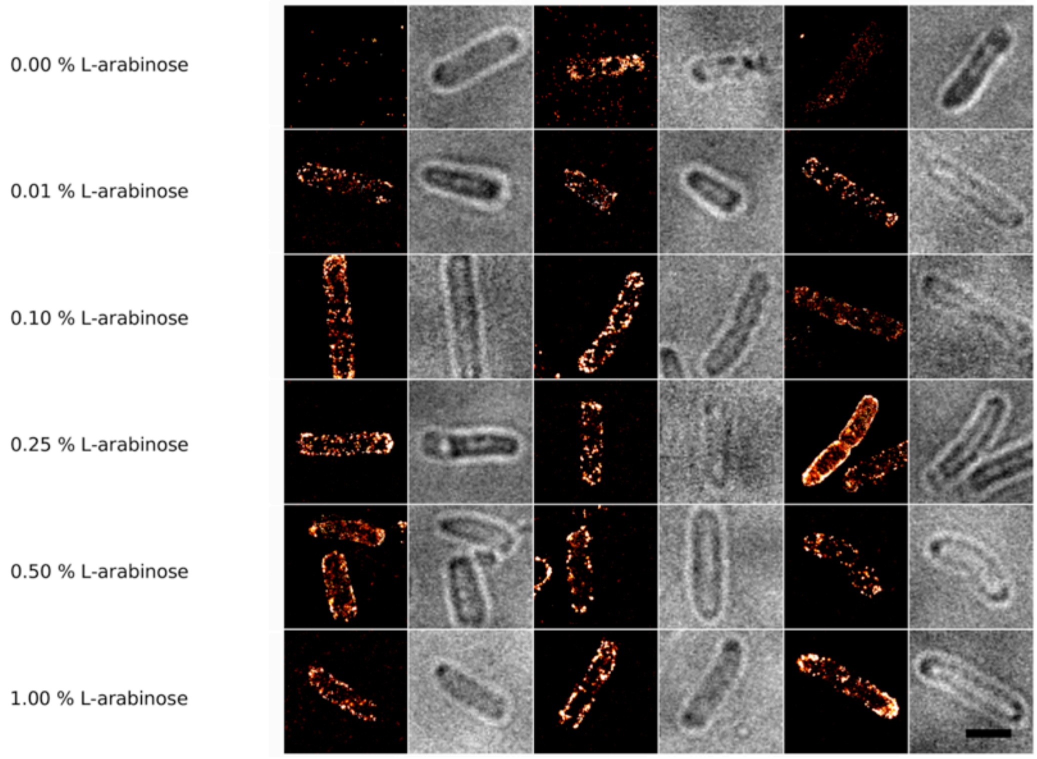

Fig. S4. qPALM of *E. coli* MJF641 expressing MscL-mEos3.2 from pBAD plasmid at various L-arabinose concentrations. Cells were grown in LB medium at 37 °C and, after induction, suspended in isosmotic PBS to allow completion of mEos3.2 maturation for 5 h. Qualitatively similar images and clustering of MscL-mEos3.2 were obtained after 1h of induction, that is after the majority of the protein had matured (see Fig. S7). Scale bar: 2 μm.

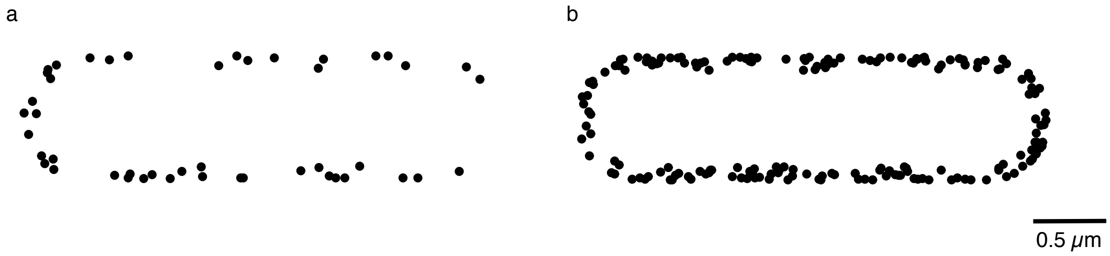

Fig. S5. Simulated distribution of (a) 100 or (b) 400 particles freely diffusing along the cell membrane of a cell with length of 3μm and a width of 0.8 μm and a hypothetical diffusion coefficient of 0.06 $\mu m^2/s$. The Smoldyn software (http://www.smoldyn.org) was used to simulate Brownian diffusion of particles for 100 s. We depict the distribution of the particles along the membrane in a field of view of 0.5 μm, which is similar to the presentation of the qPALM data.

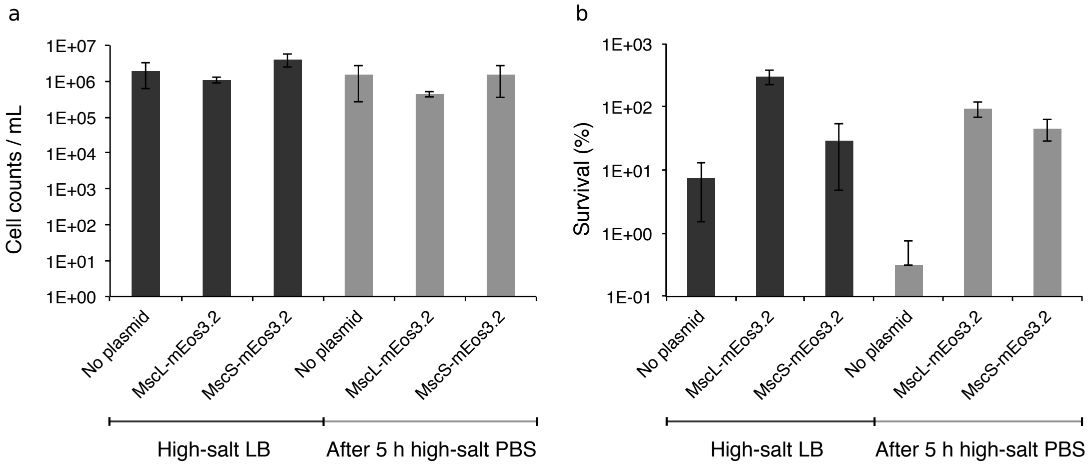

Fig. S6. Cell survival in LB and PBS. *E. coli* MJF641 adapted to high-salt LB and expressing either MscL-mEos3.2 or MscS-mEos3.2 for 1 h with 0.5% L-arabinose. (a) Cell counts per mL by colony counting on LB-agar plates directly after the induction period or after 5 h incubation in high-salt PBS. (b) Cell survival from a rapid 0.3 M NaCl downshock immediately after induction (indicated by high-salt LB) and after 5 h incubation in high-salt PBS.

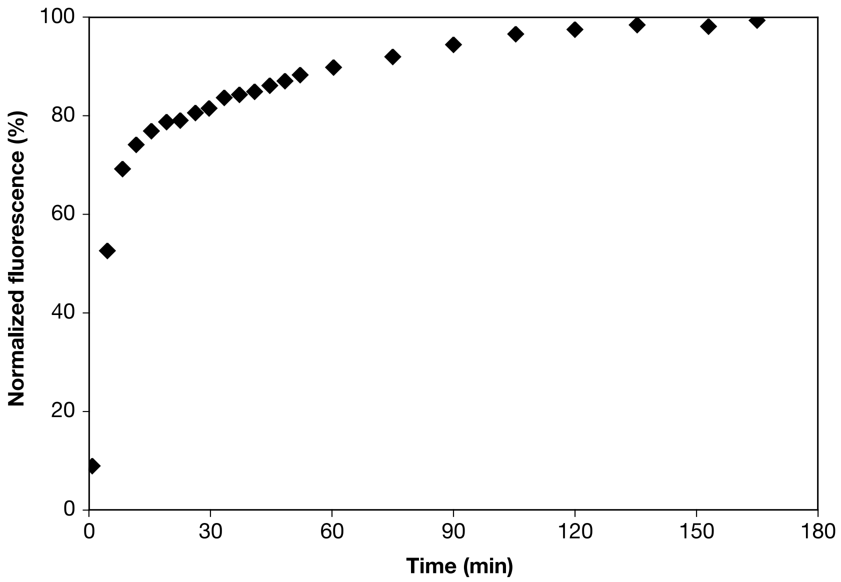

Fig. S7. *In vivo* maturation of mEos3.2. *E. coli* MC1061 expressing cytoplasmic mEos3.2 from pBAD was grown anaerobically in LB medium at 37 °C. After aeration of the culture (t = 0) the folded and non-fluorescent protein was allowed to mature. At indicated time points fluorescence was measured with flow cytometry. The fluorescence appears in two phases, one initial fast phase of maturation (and $t_{1/2} < 5$ min) and a second phase characterized by a lower rate constant ($t_{1/2}$ of 40-50 min).

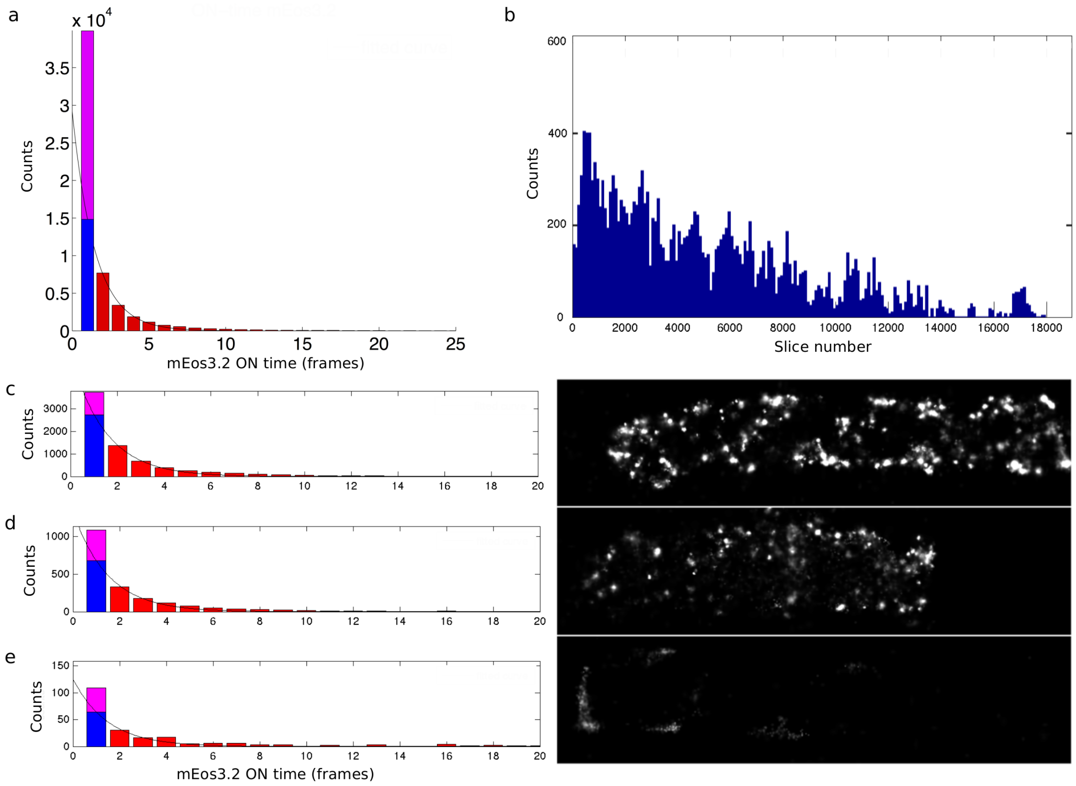

Fig. S8. *In vitro* and *in vivo* counting of mEos3.2 molecules. (a) Purified mEos3.2 molecules were immobilized on a cleaned cover slide and the ON time (t_{on}) was analysed by grouping fluorescent traces. Each frame was 31 ms. Grouped traces with a length between 2 and 19 frames (red bars) were fitted with an exponential decay and extrapolated to $x = 1$ (blue bar). The blue bar + magenta bar represent the counts that were only fluorescent for one frame. To determine the actual copy number of a fluorescent protein, the grouped traces of various lengths were summed up together with the calculated counts that only last for one frame. (b) Histogram of counts detected in *E. coli* cells expressing MscL-mEos3.2 as a function of movie acquisition time (bin size 180 counts). Towards the end of the movie there were no counts anymore, indicating that all mEos3.2 molecules were activated. (c) qPALM of MscL-mEos3.2 in *E. coli* MJF641 cells, induced with 1% (d), 0.25% and (e) 0.01% of L-arabinose. Representative cells are shown in the right panels; fluorescence counts are plotted as function of ON time (number of frames). The data were fitted to a single-exponential decay function and the counts with varying ON times were summed up to obtain the counts per cell.

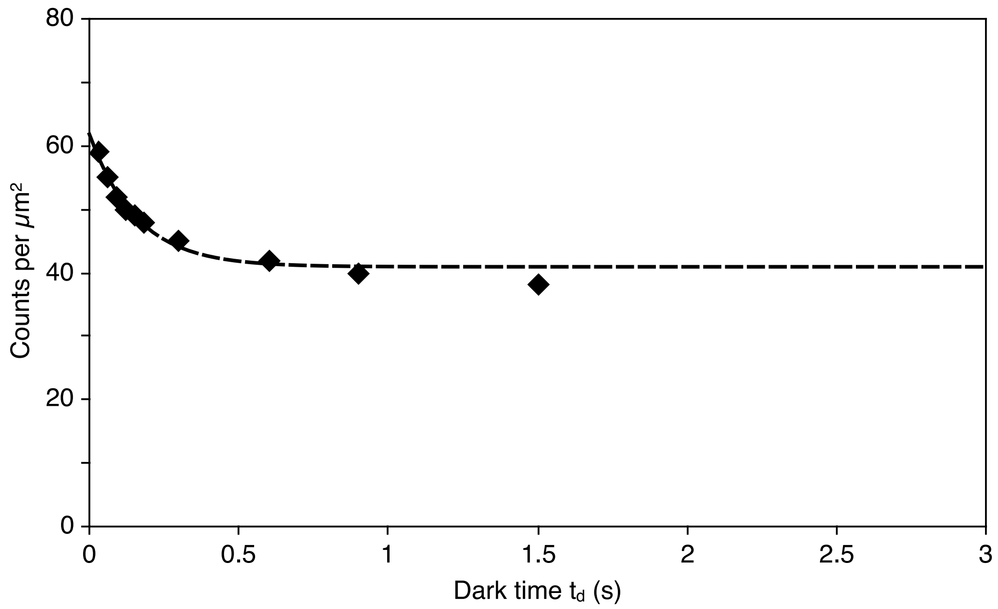

Fig. S9. Fluorescent counts *versus* dark times t_d. The fluorescence of surface-immobilized mEos3.2 was measured over time (frames of 31 ms) to quantify the blinking behaviour of mEos3.2. In order not to overestimate the real number of molecules due to blinking, we corrected for the time mEos3.2 spends in the dark state (for details of method see ref. 2). The data points can be fitted with a semi-empirical exponential function as described in Supplementary methods. The ratio of directly counted molecules and actual molecules was γ = 0.679, meaning that without correction the quantification would lead to an overestimation of around 30%.

SUPPLEMENTARY VIDEO LEGENDS

Video S1: mEos3.2-labelled MscL diffusing in the membrane of *E. coli* MJF641 at basal expression from the *trc* promoter (acquired at 30 frames per second). The channels diffuse with an average diffusion coefficient of D = 0.058 $\mu m^2/s$.

Video S2: mEos3.2-labelled MscL diffusing in the membrane of *E. coli* MJF641, induced with 0.1 mM IPTG for 30 min from the *trc* promoter (acquired at 30 frames per second). The membrane proteins are organised in clusters with an average diffusion coefficient of D = 0.0062 $\mu m^2/s$.

References

1. Zhang, M. *et al*. Rational design of true monomeric and bright photoactivatable fluorescent proteins. *Nat. Methods* **9,** 727-729 (2012).
2. Annibale, P., Vanni, S., Scarselli, M., Rothlisberger, U. & Radenovic, A. Quantitative photo activated localization microscopy: unraveling the effects of photoblinking. *PloS One* **6,** e22678 (2011).
3. Ulbrich, M. H. & Isacoff, E. Y. Subunit counting in membrane-bound proteins. *Nat. Methods* **4,** 319-321 (2007).
4. Edwards, M. D. *et al*. Characterization of three novel mechanosensitive channel activities in Escherichia coli. *Channels* **6,** 272-281 (2012).
5. Blattner, F. R. *et al*. The complete genome sequence of Escherichia coli K-12. *Science* **277,** 1453-1462 (1997).
6. Levina, N. *et al*. Protection of Escherichia coli cells against extreme turgor by activation of MscS and MscL mechanosensitive channels: identification of genes required for MscS activity. *EMBO J.* **18,** 1730-1737 (1999).
7. Casadaban, M. J. & Cohen, S. N. Analysis of gene control signals by DNA fusion and cloning in Escherichia coli. *J. Mol. Biol.* **138,** 179-207 (1980).
8. Miller, S. *et al*. Domain organization of the MscS mechanosensitive channel of Escherichia coli. *EMBO J.* **22,** 36-46 (2003).
9. Geertsma, E. R., Groeneveld, M., Slotboom, D. J. & Poolman, B. Quality control of overexpressed membrane proteins. *Proc. Natl. Acad. Sci. U. S. A.* **105,** 5722-5727 (2008).
